# Linking HLA class II susceptibility to HSPC transcriptomics and antigen presentation in aplastic anemia

**DOI:** 10.3389/fimmu.2026.1896015

**Published:** 2026-09-08

**Authors:** Maximiliano Correa Lara, Jaime García Chavez, Erika Martinez Hernandez

**Affiliations:** 1 Center for Research and Advanced Studies of the National Polytechnic Institute – Cinvestav, Molecular Biomedicine, Mexico City, Mexico; 2 National Medical Center “La Raza”, Mexican Social Security Institute, Mexico City, Mexico

**Keywords:** aplastic anemia, autoantigen, hematopoietic stem cell, human leukocitary antigens, transcriptomic analysis

## Abstract

**Background:**

Acquired aplastic anemia (AA) is an immune-mediated bone marrow failure syndrome characterized by destruction of hematopoietic stem and progenitor cells (HSPCs). Although HLA class II alleles are consistently associated with disease susceptibility, the candidate self-peptides presented by these molecules remain largely unknown.

**Methods:**

We performed a retrospective case-control study evaluating HLA class II associations in 35 patients with AA and 570 unrelated healthy controls. Bulk transcriptomic analysis of untreated Lin^-^CD34^+^ HSPCs (GSE165870) identified differentially expressed genes, which were subsequently prioritized using an independent single-cell RNA sequencing dataset (GSE145668) to identify genes preferentially expressed in HSPCs relative to T cells. Canonical protein sequences were fragmented into overlapping 15-mer peptides and analyzed for binding to disease-associated HLA class II molecules using NetMHCIIpan v4.3j.

**Results:**

HLA-DRB1*15:01, HLA-DQB1*06:02, and HLA-DRB5*01:01 were significantly enriched in patients with AA, and the three-allele co-occurrence pattern showed the strongest association with disease susceptibility. Bulk transcriptomic analysis identified 98 differentially expressed genes, including a prominent interferon-associated transcriptional program. Integration with single-cell transcriptomic data prioritized nine HSPC-enriched genes (SNCA, UHRF1, SH2D3C, SLC25A39, ADIPOR1, BNIP3L, LAT2, MEF2A, and LGALS1) for downstream analyses. NetMHCIIpan identified multiple predicted HLA class II binders across the three disease-associated HLA molecules, with SNCA, UHRF1, SH2D3C, ADIPOR1, and SLC25A39 emerging as the highest-priority candidate proteins based on normalized binding density and predicted binding affinity.

**Conclusions:**

This integrative analysis combines HLA genetics, transcriptomics, and computational peptide-binding prediction to prioritize candidate HSPC-derived self-peptides in acquired AA. These findings provide a hypothesis-generating framework for future immunopeptidomic and functional studies aimed at identifying the antigenic targets underlying immune-mediated bone marrow failure.

## 1 Introduction

Aplastic anemia (AA) is a bone marrow failure syndrome characterized by peripheral cytopenias and hypocellular marrow resulting from the depletion of hematopoietic stem and progenitor cells (HSPCs) (1). In most cases, acquired AA is driven by an immune mediated process in which autoreactive cytotoxic T cells induce apoptosis of hematopoietic progenitors, leading to progressive marrow failure (1, 2). This immune pathophysiology is supported by clinical responses to immunosuppressive therapy and by experimental observations demonstrating oligoclonal T cell expansion, increased interferon γ signaling, and reduced regulatory T-cell populations in affected patients (1–3).

Genetic studies have consistently implicated the human leukocyte antigen (HLA) region in susceptibility to AA. Among these, HLA-DRB1*15:01 and the extended DR15 haplotype, including HLA-DQB1*06:02, represent the most reproducible risk alleles across multiple populations and have also been associated with response to immunosuppressive therapy. These observations suggest that disease susceptibility may be influenced by HLA class II-restricted presentation of self-peptides to autoreactive CD4^+^ T cells. However, despite compelling epidemiological evidence linking HLA polymorphisms to AA, the identity of the peptides presented by these disease-associated HLA molecules has remained elusive (4–6).

Previous attempts to identify candidate autoantigens in AA have primarily relied on serological screening or peptide-specific T-cell assays. Hirano et al. identified kinectin as a candidate autoantigen through autoantibody screening and demonstrated that kinectin-derived peptides could suppress hematopoietic colony formation *in vitro*, although naturally occurring kinectin-specific T cells were not identified in patients (7). Similarly, Feng et al. identified diazepam-binding inhibitor-related protein 1 (DRS-1) as an HLA-DR15-restricted candidate antigen capable of stimulating peptide-specific T-cell responses in a limited number of patients (8). Although these studies provided important proof-of-principle evidence supporting antigen-driven immunity in AA, candidate proteins were identified independently of disease-associated HSPC transcriptomic alterations and without systematic prioritization according to HLA genotype or cell-type–specific expression.

Recent transcriptomic studies have substantially advanced our understanding of the molecular landscape of AA. Single-cell RNA sequencing has demonstrated that residual HSPCs exhibit profound lineage-specific transcriptional alterations, chronic interferon signaling, dysregulated RNA processing, and altered interactions with infiltrating T cells, highlighting the persistent inflammatory state of the target hematopoietic compartment. Furthermore, genomic studies have shown that immune pressure in AA promotes clonal selection through alterations affecting HLA genes and other components of the antigen presentation machinery, reinforcing the concept that HLA-mediated antigen presentation plays a central role in disease pathogenesis (9, 10). Despite these advances, no study has systematically integrated disease-associated HLA genetics with transcriptomic alterations in HSPCs to prioritize candidate self-peptides capable of binding AA-associated HLA class II molecules.

To address this gap, we integrated HLA association analysis with publicly available transcriptomic datasets to prioritize HSPC-derived proteins capable of generating peptides predicted to bind disease-associated HLA class II molecules. Rather than establishing a direct HLA–antigen mechanism, our study provides a hypothesis-generating framework for prioritizing candidate self-antigens for future immunopeptidomic and functional validation.

## 2 Materials and methods

### 2.1 Study design and cohort definition

This retrospective case-control study investigated the association between HLA class II alleles and susceptibility to acquired aplastic anemia (AA) and integrated these findings with public transcriptomic datasets to prioritize candidate HSPC-derived self-peptides. The study was conducted in accordance with the Declaration of Helsinki and approved by the Institutional Ethics and Research Committees of the Instituto Mexicano del Seguro Social (IMSS), UMAE Hospital de Especialidades “Dr. Antonio Fraga Mouret,” Centro Médico Nacional “La Raza” (protocol R-2019-3501-020) (11).

Thirty-five patients diagnosed with acquired AA according to established international diagnostic criteria were included (1). The control group consisted of 570 unrelated volunteer donors who underwent high-resolution HLA typing at the same institution. Related donors were excluded to minimize genetic bias.

### 2.2 HLA typing and association analysis

High-resolution HLA typing was performed using next-generation sequencing (Illumina MiSeq) covering HLA class I and class II loci (12). Allele frequencies were analyzed using a carrier (dominant) model, in which individuals carrying at least one copy of a given allele were compared with non-carriers (13).

Associations between HLA alleles and AA were evaluated using Fisher’s exact test. Odds ratios (ORs) and 95% confidence intervals (95% CIs) were calculated from 2 × 2 contingency tables using the Haldane–Anscombe correction when zero counts were present. P values were adjusted for multiple comparisons using the Benjamini–Hochberg false discovery rate (FDR) procedure, with FDR < 0.05 considered statistically significant (14–16).

To evaluate the combined presence of the disease-associated alleles, a three-allele co-occurrence analysis was performed for HLA-DRB1*15:01, HLA-DQB1*06:02, and HLA-DRB5*01:01. Because haplotype phasing was not performed, these analyses evaluated allele co-occurrence within individuals rather than chromosomal haplotypes. Fisher’s exact test was used to compare the frequency of the three-allele combination between patients and controls.

### 2.3 Bulk transcriptomic analysis

Bulk RNA-seq data were obtained from the Gene Expression Omnibus (GEO) dataset GSE165870. Only untreated Lin^-^CD34^+^ HSPC samples corresponding to six AA patients (P18–P23) and three healthy controls (Ctrl2, Ctrl7, and Ctrl8) were included, following the experimental design of the original study. Samples generated after treatment or belonging to other experimental conditions were excluded to avoid treatment-related transcriptional effects.

Raw gene-count matrices were analyzed using DESeq2 in R (version 4.5.2). Genes with fewer than ten total counts across all samples were excluded before normalization. Differential expression analysis was performed using the Wald test implemented in DESeq2. Genes were considered differentially expressed when they fulfilled FDR < 0.05 and |log_2_ fold change| > 1 (17–19).

### 2.4 Lineage-gene filtering and single-cell transcriptomic prioritization

Because Lin^-^CD34^+^ cell preparations contain heterogeneous HSPC populations, including lineage-biased progenitors, lineage-associated transcripts were not removed from the differential expression analysis itself. Instead, canonical erythroid, lymphoid, and myeloid marker genes (**Supplementary Table 1**) were excluded only during the candidate autoantigen prioritization step to reduce the influence of lineage-restricted differentiation markers on downstream peptide prediction.

The remaining candidate genes were independently evaluated using the publicly available single-cell RNA sequencing dataset GSE145668. This dataset was analyzed exclusively as a cell-type prioritization resource rather than an independent validation cohort because it represents an independent patient population generated within the same study. Candidate genes were retained when they satisfied all of the following predefined criteria:

Mean normalized expression in HSPCs > 0.5;

Expression detected in >20% of HSPCs;

Mean expression in HSPCs greater than or equal to that observed in T cells.

These criteria were selected to prioritize genes broadly expressed within the target HSPC compartment while minimizing genes predominantly associated with mature immune-cell populations. Consequently, the retained genes are described throughout the manuscript as HSPC-enriched relative to T cells rather than HSPC-specific.

The general characteristics of the public transcriptomic datasets included in the present study are summarized in **Supplementary Table 2**.

### 2.5 Protein sequence retrieval and peptide generation

Canonical protein sequences corresponding to the HSPC-enriched candidate genes were retrieved from the UniProtKB/Swiss-Prot database using biomaRt. Only manually reviewed Swiss-Prot entries were retained (20).

Protein sequences were computationally fragmented into overlapping 15-amino acid peptides using a sliding-window approach with a one-amino-acid offset. Peptide identifiers were preserved throughout the workflow to maintain traceability between each peptide and its source protein during downstream analyses.

### 2.6 HLA class II peptide-binding prediction

Peptide binding to HLA class II molecules was predicted using NetMHCIIpan version 4.3j operating in EL+BA prediction mode (21). Binding predictions were performed for the AA-associated HLA molecules identified in the genetic association analysis, including HLA-DRB1*15:01, HLA-DRB5*01:01, and HLA-DQA101:02/DQB106:02.

All overlapping 15-mer peptides were evaluated against each HLA molecule. Peptides were classified according to the default NetMHCIIpan EL-rank thresholds as:

Strong binders: EL rank ≤1%;

Weak binders: EL rank >1% and ≤5%;

Non-binders: EL rank >5%.

For each peptide–HLA combination, the predicted binding core, EL score, EL rank, BA score, BA rank, and predicted binding affinity (nM) were extracted.

### 2.7 Prioritization of candidate HSPC-derived peptides

To compare proteins of different lengths, the number of predicted binders was normalized to the total number of overlapping peptides generated from each protein. Strong-binder density and total binder density were calculated as the proportion of strong binders or total predicted binders (strong plus weak) relative to the total number of peptides derived from the corresponding protein.

For each protein–HLA combination, the peptide exhibiting the lowest EL rank was selected as the representative candidate epitope. These representative peptides were used for comparative analyses and graphical visualization.

Data manipulation and visualization were performed using the dplyr, tidyr, ggplot2, patchwork, readxl, openxlsx, Biostrings, stringr, org.Hs.eg.db, and biomaRt packages (22–24).

## 3 Results

### 3.1 Study population and cohort characteristics

The study included 35 patients with acquired aplastic anemia (AA) and 570 unrelated healthy donors with available high-resolution HLA genotyping after exclusion of related donors; The clinical and demographic characteristics of the AA cohort are summarized in **Table 1**.

**Table 1:** Clinical and demographic characteristics of the AA cohort.

Characteristic	Acquired aplastic anemia (n = 35)	Healthy controls (n = 570)
Age, years, median (IQR)	30.0 (21.5–43.5)	Not systematically available
Sex	17 female (48.6%), 18 male (51.4%)	224 female (39.3%), 346 male (60.7%)
Disease severity (Camitta criteria)		—
• Non-severe AA	4 (11.4%)	—
• Severe AA	27 (77.1%)	—
• Very severe AA	4 (11.4%)	—
Previous immunosuppressive therapy		—
• Cyclosporine	35 (100%)	—
• Eltrombopag	34 (97.1%)	—
• Antithymocyte globulin (≥1 course)	24 (68.6%)	—
• Repeat ATG course	8 (22.9%)	—
Documented PNH clone	9 (25.7%)	—
Reported cytogenetic abnormalities	3 (8.6%)	—
Bone marrow hypocellularity	35 (100%)	—
Institution	Hospital de Especialidades, CMN La Raza, IMSS	Same institution
Ethnicity	Mexican	Mexican
HLA typing	High-resolution next-generation sequencing (NGS)	High-resolution next-generation sequencing (NGS)

Carrier-based association analysis identified a significant enrichment of HLA-DRB1*15:01 among AA patients compared with healthy controls (10/35 [28.6%] vs. 31/570 [5.4%]; OR = 6.95, 95% CI 3.07–15.76; P = 3.58 × 10^-5^; FDR q = 0.00161) (**Table 2**). Although HLA-DRB114:06 was absent among AA patients and detected in 76/570 (13.3%) healthy controls, this association did not remain statistically significant after correction for multiple testing (OR = 0.09, 95% CI 0.01–1.50; P = 0.0151; FDR q = 0.319). Likewise, HLA-DRB116:02, HLA-DRB104:07, HLA-DRB108:02, and all remaining HLA-DRB1 alleles showed no significant association after false discovery rate correction.

**Table 2:** Most relevant HLA associations.

Genetic marker	AA N/N (%)	Controls N/N (%)	OR	95% CI	Raw p-value	FDR (q)
DRB1*15:01	10/35 (28.6%)	31/570 (5.4%)	6.95	3.07–15.76	3.58×10^-5^	0.00161
DRB1*14:06	0/35 (0.0%)	76/570 (13.3%)	0.09	0.01–1.50	0.0151	0.319
DRB1*04:07	15/35 (42.9%)	180/570 (31.6%)	1.62	0.81–3.25	0.192	1.000
DRB1*16:02	6/35 (17.1%)	58/570 (10.2%)	1.83	0.73–4.58	0.249	1.000
DRB1*08:02	11/35 (31.4%)	190/570 (33.3%)	0.92	0.44–1.91	1.000	1.000

Given the established association between HLA-DRB1*15:01 and the canonical DR2 susceptibility background, additional analyses focused on the co-occurrence of HLA-DRB1*15:01, HLA-DQB1*06:02, and HLA-DRB5*01:01 (**Table 3**). HLA-DQB106:02 was significantly enriched in AA patients (10/35 [28.6%] vs. 28/570 [4.9%]; OR = 7.74, 95% CI 3.39–17.68; P = 1.70 × 10^-5^; FDR q = 5.11 × 10^-5^), as was HLA-DRB501:01 (9/35 [25.7%] vs. 34/570 [6.0%]; OR = 5.46, 95% CI 2.37–12.56; P = 3.53 × 10^-4^; FDR q = 3.53 × 10^-4^). In addition, the HLA-DQ α-chain allele HLA-DQA1*01:02, which forms the canonical DQA101:02–DQB106:02 heterodimer, was also significantly associated with AA (9/35 [25.7%] vs. 57/570 [10.0%]; OR = 3.12, 95% CI 1.39–6.97; P = 0.0088).

**Table 3:** DR2-associated alleles and haplotype.

Marker	Definition	AA N/N (%)	Controls N/N (%)	OR	95% CI	P-value	FDR (q)
DRB1*15:01	Carrier	10/35 (28.6%)	31/570 (5.4%)	6.95	3.07–15.76	3.6×10^-5^	5.4×10^-5^
DQB1*06:02	Carrier	10/35 (28.6%)	28/570 (4.9%)	7.74	3.39–17.68	1.7×10^-5^	5.1×10^-5^
DRB5*01:01	Carrier	9/35 (25.7%)	34/570 (6.0%)	5.46	2.37–12.56	3.5×10^-4^	3.5×10^-4^
DR2 haplotype	DRB1*15:01 + DQB1*06:02 + DRB5*01:01	9/35 (25.7%)	21/570 (3.7%)	9.05	3.29–23.02	1.5×10^-5^	—

Evaluation of the co-occurrence of HLA-DRB1*15:01, HLA-DQB1*06:02, and HLA-DRB5*01:01 demonstrated that simultaneous carriage of these three canonical DR2 susceptibility alleles was significantly more frequent among AA patients than healthy controls (9/35 [25.7%] vs. 21/570 [3.7%]; OR = 9.05, 95% CI 3.29–23.02; P = 1.53 × 10^-5^) (**Table 3**). Because phase information was unavailable, this analysis represents carrier co-occurrence of the canonical DR2 susceptibility alleles rather than direct haplotype inference.

### 3.2 Bulk transcriptomic analysis identifies an interferon-associated transcriptional program in AA-derived HSPCs

**Figure 1:**
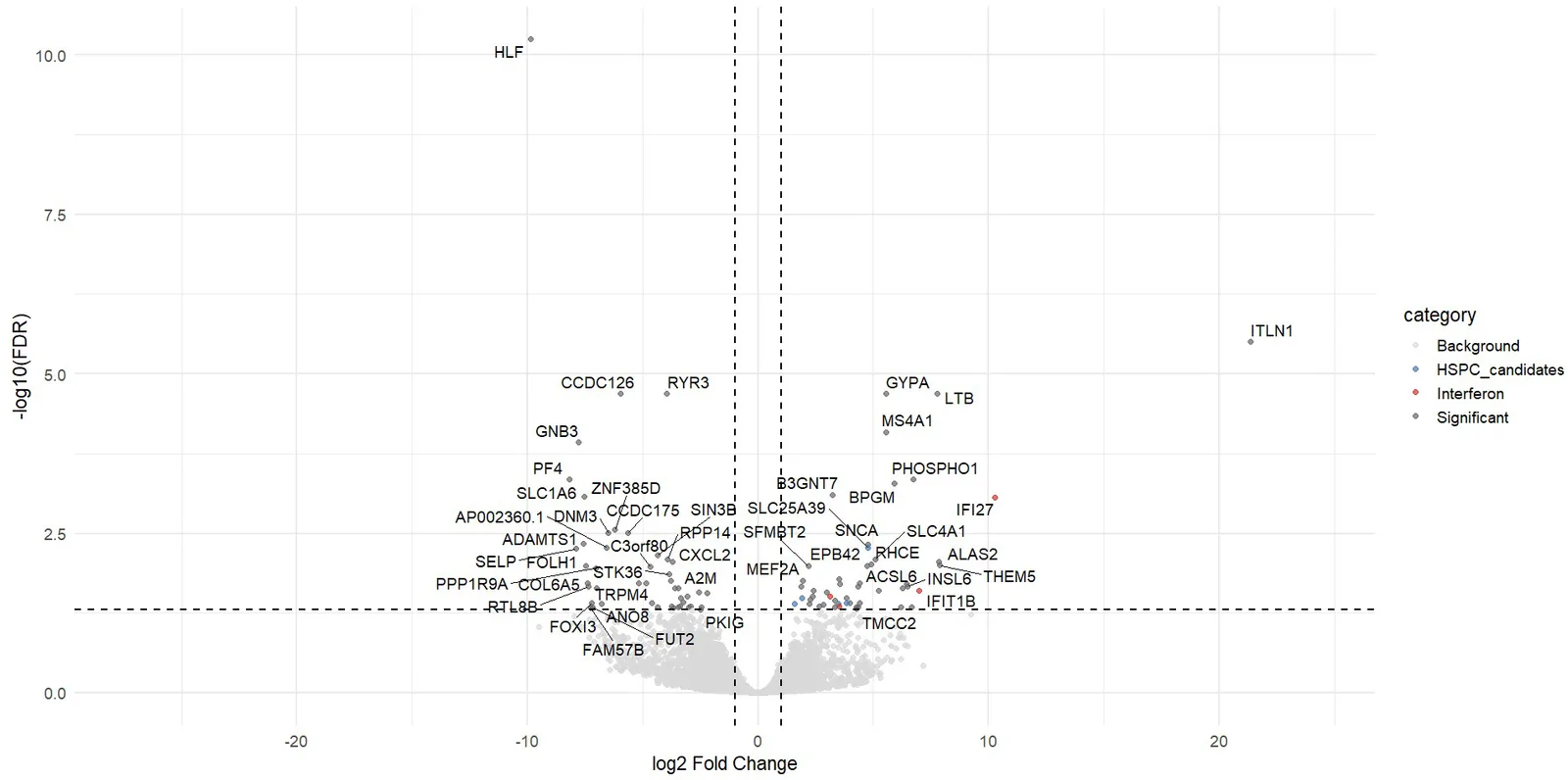
Bulk transcriptomic analysis identifies an interferon-associated transcriptional program and candidate HSPC-derived proteins in acquired aplastic anemia. Volcano plot showing differential gene expression in Lin^-^CD34^+^ hematopoietic stem and progenitor cells (HSPCs) from untreated patients with acquired aplastic anemia (AA; n = 6) compared with healthy controls (n = 3) using the GSE165870 dataset. Differentially expressed genes were identified using DESeq2 with thresholds of FDR < 0.05 and |log_2_ fold change| > 1. Vertical dashed lines indicate the log_2_ fold-change cutoff (± 1), and the horizontal dashed line indicates the statistical significance threshold (FDR = 0.05). Interferon-responsive genes are highlighted in red, HSPC-enriched candidate genes selected for downstream prioritization are shown in blue, other significant differentially expressed genes are shown in dark gray, and non-significant genes are shown in light gray. Candidate HSPC-derived proteins were subsequently prioritized through single-cell transcriptomic analysis and HLA class II peptide-binding prediction.

To characterize transcriptional alterations within the target hematopoietic compartment, bulk RNA sequencing data from untreated Lin^-^CD34^+^ HSPCs (GSE165870) were analyzed. Differential expression analysis identified 98 significantly deregulated genes (FDR < 0.05 and |log_2_FC| > 1), comprising 51 upregulated and 47 downregulated transcripts. The most significantly upregulated genes included IFI27 (log_2_FC = 10.28, FDR = 8.35 × 10^-4^), IFIT1B (log_2_FC = 7.00, FDR = 2.42 × 10^-2^), IFIT2 (log_2_FC = 3.51, FDR = 4.26 × 10^-2^), and IRF7 (log_2_FC = 3.13, FDR = 2.96 × 10^-2^). (**Figure 1**); The complete DESeq2 results is provided in **Supplementary Table 3**.

### 3.3 Single-cell transcriptomic prioritization identifies HSPC-enriched candidate proteins

To prioritize proteins intrinsically expressed within the target HSPC compartment, candidate genes identified by bulk RNA sequencing were independently evaluated using the single-cell RNA sequencing dataset GSE145668. Because this dataset represents an independent patient cohort, it was used as a cell-type prioritization resource rather than an independent validation of disease-associated differential expression.

**Figure 2:**
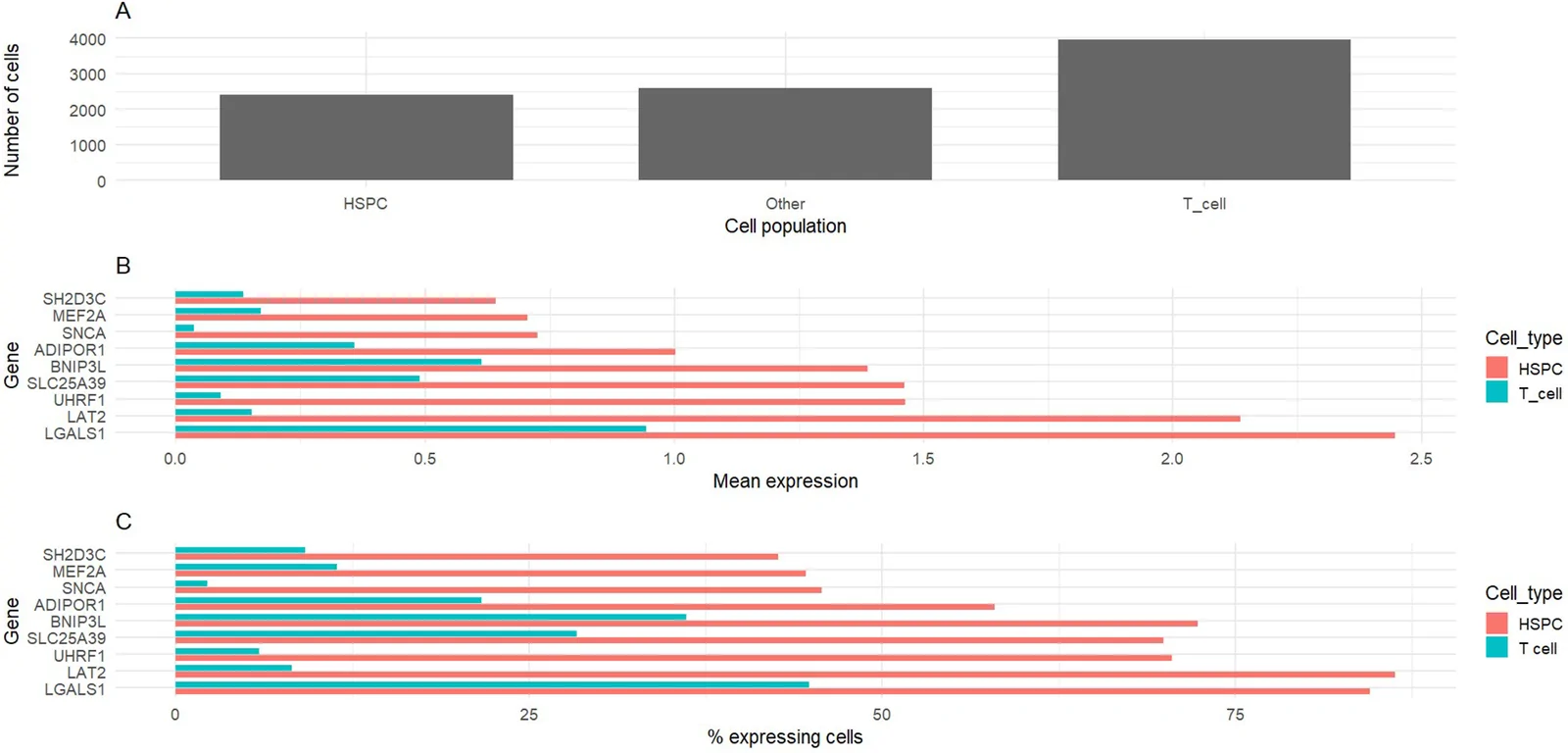
Single-cell validation of HSPC-specific candidate gene expression. **(A)** Distribution of cells across the three major cell populations identified in the GSE145668 dataset after cell-type annotation, including hematopoietic stem and progenitor cells (HSPCs), T cells, and other bone marrow cell populations. **(B)** Mean normalized expression of the nine candidate genes identified from the bulk RNA-seq analysis in HSPCs and T cells. Genes were retained for downstream analyses when they demonstrated preferential expression within the HSPC compartment relative to T cells. **(C)** Percentage of HSPCs and T cells expressing each candidate gene. Candidate genes were prioritized based on predefined criteria requiring detectable expression (mean normalized expression >0.5), expression in at least 20% of HSPCs, and expression levels equal to or greater than those observed in T cells. This analysis was performed to prioritize HSPC-enriched relative to T-cell transcripts before HLA class II peptide-binding prediction and was not intended to validate disease-associated differential expression.

Candidate genes were retained when they exhibited (i) mean HSPC expression >0.5, (ii) expression in >20% of HSPCs, and (iii) expression in HSPCs greater than or equal to that observed in T cells. Application of these predefined criteria identified nine HSPC-enriched genes relative to T cells, including SNCA, LAT2, UHRF1, SH2D3C, SLC25A39, ADIPOR1, BNIP3L, MEF2A, and LGALS1 (**Figure 2**, **Table 4**). These genes displayed log_2_ fold changes ranging from 1.59 to 4.78, HSPC-to-T-cell expression ratios ranging from 2.27 to 20.45 and were detected in 42.7%–86.3% of HSPCs (**Table 4**).

**Table 4:** HSPC-enriched candidate autoantigen genes identified by integrated bulk and single-cell transcriptomic analyses.

Gene	log2FC	FDR	HSPC expression	T-cell expression	HSPC/T-cell ratio	% HSPCs expressing	% T cells expressing
SNCA	4.78	0.0047	0.73	0.04	20.45	45.7	2.2
LAT2	1.88	0.0218	2.14	0.15	14.08	86.3	8.2
UHRF1	1.90	0.0329	1.46	0.09	16.26	70.5	5.9
SH2D3C	2.23	0.0401	0.64	0.14	4.74	42.7	9.2
SLC25A39	4.76	0.0053	1.46	0.49	2.99	69.9	28.4
ADIPOR1	3.83	0.0391	1.00	0.36	2.81	57.9	21.7
BNIP3L	3.48	0.0401	1.39	0.61	2.27	72.4	36.1
MEF2A	1.95	0.0174	0.71	0.17	4.15	44.6	11.4
LGALS1	1.59	0.0399	2.45	0.94	2.60	84.5	44.8

### 3.4 HLA class II binding prediction identifies multiple HSPC-derived candidate self-peptides

Canonical protein sequences corresponding to the nine HSPC-enriched genes were fragmented into 3,505 overlapping 15-mer peptides, which were evaluated against HLA-DRB1*15:01, HLA-DRB5*01:01, and HLA-DQA101:02/DQB106:02 using NetMHCIIpan version 4.3j. The complete cleaned prediction output is provided in **Supplementary Table 4**, while protein sequence information, UniProt identifiers, sequence lengths, and peptide generation details are summarized in **Supplementary Table 5**.

**Figure 3:**
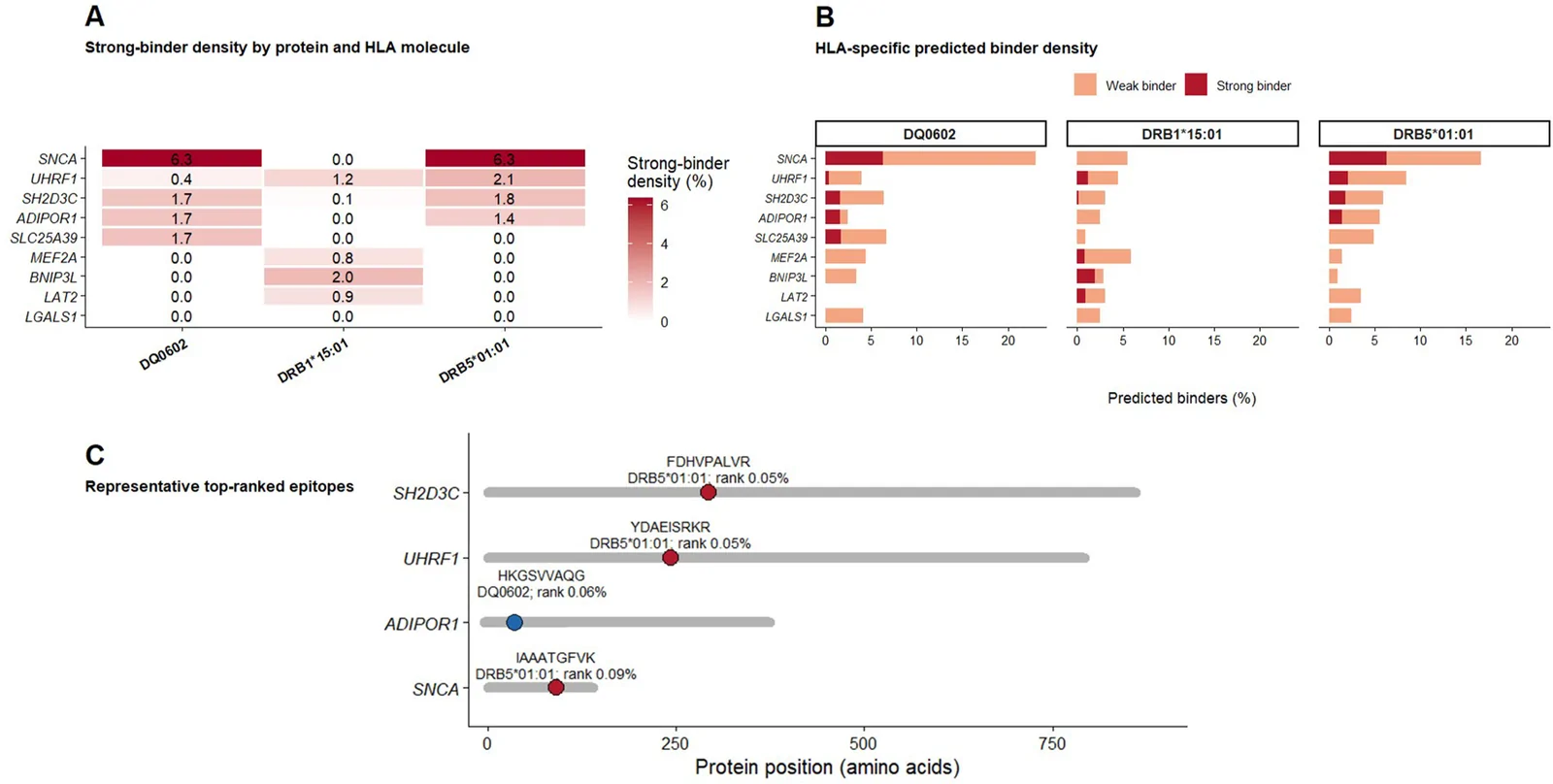
Integrated prioritization of candidate HSPC-derived autoantigens. **(A)** Heatmap showing the density of predicted strong-binding peptides derived from nine HSPC-enriched proteins across HLA-DQA101:02/DQB106:02, HLA-DRB115:01, and HLA-DRB501:01. Strong-binder density was calculated as the percentage of overlapping 15-mer peptides from each protein with a NetMHCIIpan EL rank ≤1% for the indicated HLA molecule. **(B)** Density of predicted strong and weak binders across all three evaluated HLA molecules. Weak binders were defined as peptides with an EL rank >1% and ≤5%. **(C)** Linear representation of selected source proteins showing the locations of representative top-ranked 15-mer peptides and their predicted 9-amino-acid binding cores.

A total of 10,515 peptide–HLA combinations were evaluated. NetMHCIIpan predicted 37 strong and 143 weak binders for HLA-DQA101:02/DQB106:02, 20 strong and 105 weak binders for HLA-DRB1*15:01, and 44 strong and 150 weak binders for HLA-DRB5*01:01 (**Figures 3A, B**).

To account for differences in protein length, binding predictions were normalized to the total number of overlapping peptides generated from each protein (**Table 5**). SNCA exhibited the highest density of predicted strong binders (4.23%) and the highest total binder density (15.08%), followed by UHRF1 (1.20% and 5.65%, respectively), SH2D3C (1.18% and 5.12%), and ADIPOR1 (1.02% and 3.51%). No predicted strong binders were identified for LGALS1.

**Table 5:** Summary of NetMHCIIpan predictions for HSPC-derived proteins.

Protein	15-mers generated	Strong binders	Weak binders	Strong binder density (%)	Total binder density (%)
SNCA	126	16	41	4.23	15.08
UHRF1	779	28	104	1.20	5.65
SH2D3C	846	30	100	1.18	5.12
ADIPOR1	361	11	27	1.02	3.51
BNIP3L	205	4	11	0.65	2.44
SLC25A39	345	6	37	0.58	4.15
LAT2	229	2	13	0.29	2.18
MEF2A	493	4	54	0.27	3.92
LGALS1	121	0	11	0.00	3.03

HLA-specific analyses demonstrated distinct binding profiles among the three disease-associated HLA molecules. SNCA exhibited the highest strong-binder density for HLA-DQA101:02/DQB106:02 (6.35%), whereas UHRF1 (2.05%) and SH2D3C (1.77%) showed the highest densities for HLA-DRB5*01:01. For HLA-DRB1*15:01, the highest strong-binder density was observed for BNIP3L (1.95%), followed by UHRF1 (1.16%) and LAT2 (0.87%) (**Figures 3A, B**).

### 3.5 Identification of representative candidate HLA class II epitopes

For each protein–HLA combination, the peptide with the lowest predicted EL rank was selected as the representative candidate epitope (**Table 6**). The highest-confidence predictions included EQESFDHVPALVRYH derived from SH2D3C (HLA-DRB5*01:01, EL rank = 0.05%, predicted affinity = 14.6 nM), GFWYDAEISRKRETR derived from UHRF1 (HLA-DRB5*01:01, EL rank = 0.05%, affinity = 39.3 nM), and MSSHKGSVVAQGNGA derived from ADIPOR1, containing the predicted binding core HKGSVVAQG, for HLA-DQA101:02/DQB106:02 (EL rank = 0.06%, affinity = 125.5 nM). Representative top-ranked epitopes derived from SNCA, UHRF1, SH2D3C, and ADIPOR1 are illustrated in **Figure 3C**, demonstrating that multiple HSPC-derived proteins contain peptides predicted to bind AA-associated HLA class II molecules.

**Table 6:** Highest-confidence predicted HLA-II epitopes.

Gene	HLA molecule	15-mer peptide	Binding core	El rank (%)	Affinity (nM)	Binding
SH2D3C	HLA-DRB5*01:01	EQESFDHVPALVRYH	FDHVPALVR	0.05	14.6	Strong
UHRF1	HLA-DRB5*01:01	GFWYDAEISRKRETR	YDAEISRKR	0.05	39.3	Strong
ADIPOR1	HLA-DQA1*01:02/DQB1*06:02	MSSHKGSVVAQGNGA	HKGSVVAQG	0.06	125.5	Strong
SNCA	HLA-DRB5*01:01	AGSIAAATGFVKKDQ	IAAATGFVK	0.09	25.6	Strong
UHRF1	HLA-DQA1*01:02/DQB1*06:02	ECRNDASEVVLAGER	NDASEVVLA	0.10	48.7	Strong
SLC25A39	HLA-DQA1*01:02/DQB1*06:02	APMVAGALARLGTVT	VAGALARLG	0.12	26.8	Strong
SNCA	HLA-DQA1*01:02/DQB1*06:02	QVTNVGGAVVTGVTA	NVGGAVVTG	0.14	64.7	Strong
SH2D3C	HLA-DQA1*01:02/DQB1*06:02	GSTEHGVEVVLAHLE	EHGVEVVLA	0.16	82.5	Strong

## 4 Discussion

In the present study, we integrated HLA association analysis with bulk transcriptomics, single-cell transcriptomic prioritization, and HLA class II peptide-binding prediction to systematically prioritize candidate HSPC-derived self-peptides. Importantly, our study was designed as a candidate-prioritization framework rather than a direct demonstration of antigen presentation. Because the HLA cohort, bulk transcriptomic dataset, and single-cell RNA-seq dataset originated from independent patient populations, the results should be interpreted as cross-cohort evidence integration, providing biologically plausible candidates for future functional validation rather than establishing patient-level HLA–antigen relationships.

Consistent with previous studies, we observed a strong enrichment of HLA-DRB1*15:01, HLA-DQB1*06:02, and HLA-DRB5*01:01 among patients with AA. The association of the DR15-related alleles with disease susceptibility has been repeatedly reported across different populations and supports the central role of HLA class II–restricted immunity in AA pathogenesis (5, 6). Previous work has suggested that these alleles may influence the presentation of self-peptides capable of activating autoreactive CD4^+^ T cells, although the relevant antigenic targets have remained unknown (7, 8). Our findings reinforce this concept by identifying multiple HSPC-derived proteins predicted to generate peptides with high binding affinity for these disease-associated HLA molecules.

Bulk transcriptomic analysis of untreated Lin^-^CD34^+^ HSPCs revealed a prominent interferon-associated transcriptional program characterized by marked upregulation of IFI27, IFIT1B, IFIT2, and IRF7. These findings closely mirror the transcriptional landscape previously reported by Zhu and colleagues, who demonstrated widespread activation of interferon signaling and inflammatory pathways within residual HSPCs in AA (10). Rather than representing candidate autoantigens themselves, these interferon-responsive genes likely reflect the chronic inflammatory environment created by activated T cells and myelosuppressive cytokines (2). Accordingly, our prioritization strategy focused on proteins that were both differentially expressed and preferentially represented within the HSPC compartment.

A major strength of the present study is the incorporation of single-cell transcriptomic data as a cell-type prioritization step. Unlike previous studies that used single-cell RNA sequencing to characterize disease-associated transcriptional changes, we used the independent GSE145668 dataset to determine whether candidate genes identified by bulk RNA sequencing were preferentially expressed within HSPCs relative to T cells. This approach was not intended to validate differential expression between AA and healthy HSPCs, but rather to reduce the likelihood that downstream peptide predictions would be driven by genes predominantly expressed in infiltrating immune cells or lineage-restricted progenitors. The resulting set of nine HSPC-enriched genes provided a biologically informed starting point for downstream HLA class II peptide prediction.

Rather than identifying a single dominant antigen, our analysis demonstrated that several proteins generated peptides predicted to bind disease-associated HLA class II molecules. SNCA exhibited the highest normalized density of predicted strong binders, whereas UHRF1 and SH2D3C generated the strongest predicted ligands for HLA-DRB5*01:01. ADIPOR1 remained one of the highest-ranking candidates for HLA-DQA101:02/DQB106:02, but the expanded analysis indicates that it should be considered part of a broader repertoire of candidate self-antigens rather than the unique target of the autoimmune response. This observation is biologically plausible because autoimmune diseases rarely depend on a single immunodominant antigen and instead frequently involve multiple intracellular proteins capable of generating immunogenic peptides.

Our findings also complement recent work by Pagliuca et al., who demonstrated that immune pressure in AA shapes the genomic landscape of HSPCs through selective disruption of antigen presentation pathways and acquisition of immune escape mechanisms (25). That study showed that HLA class I and class II genes themselves become targets of somatic alterations during disease evolution, supporting the concept that antigen presentation is central to AA pathophysiology. Our results extend this model by identifying HSPC-derived proteins predicted to generate peptides for those same disease-associated HLA molecules. Although direct evidence of endogenous peptide presentation is still lacking, the combination of HLA susceptibility, HSPC-restricted expression, and predicted HLA binding provides a rational framework for prioritizing candidate self-antigens for future investigation.

Several limitations should be acknowledged. First, the HLA cohort, bulk transcriptomic data, and single-cell RNA-seq data were obtained from independent cohorts, precluding direct patient-level integration of HLA genotype, gene expression, and peptide presentation. Second, NetMHCIIpan predicts peptide–HLA binding affinity but does not demonstrate endogenous peptide processing, presentation on the cell surface, or recognition by autoreactive T cells. Third, the single-cell dataset was used to prioritize genes preferentially expressed within the HSPC compartment rather than to validate disease-associated differential expression. Finally, the candidate peptides identified here require experimental confirmation using complementary approaches such as HLA ligand elution, mass spectrometry–based immunopeptidomics, peptide-specific CD4^+^ T-cell assays, and functional studies in patients carrying the corresponding HLA alleles.

## 5 Conclusion

In conclusion, this study provides an integrative framework for prioritizing candidate HSPC-derived self-peptides in acquired aplastic anemia by combining disease-associated HLA genetics, bulk transcriptomics, single-cell cell-type prioritization, and HLA class II peptide-binding prediction. Rather than identifying a single dominant autoantigen, our findings support a model in which multiple HSPC-derived proteins including SNCA, UHRF1, SH2D3C, ADIPOR1, and SLC25A39 constitute a repertoire of candidate HLA-binding self-antigens for future experimental validation. This workflow provides a systematic strategy for narrowing the search for pathogenic autoantigens while remaining consistent with current evidence that immune-mediated marrow failure is likely driven by a complex network of HLA-restricted autoreactive responses rather than a single antigenic target.

## Data Availability

The datasets presented in this study can be found in online repositories. The names of the repository/repositories and accession number(s) can be found in the article/**Supplementary Material**.
