# Supplementary material for: Linking HLA class II susceptibility to HSPC transcriptomics and antigen presentation in aplastic anemia
Source: Front Immunol. 2026 Sep 8;17:1896015. doi: 10.3389/fimmu.2026.1896015 (PMC13593780; doi:10.3389/fimmu.2026.1896015)
Supplement: Supplementary file 1 [file Table1.docx]

**Suplemental material**

**Supplementary Table S1. Canonical lineage-associated genes excluded during candidate autoantigen prioritization**

| Gene | Predominant lineage | Biological function | Rationale for exclusion | Supporting reference |
| --- | --- | --- | --- | --- |
| HBB | Erythroid | Hemoglobin subunit β | Canonical marker of erythroid differentiation | Orkin & Zon, *Cell* (2008) [1]. |
| HBA1 | Erythroid | Hemoglobin subunit α1 | Canonical marker of erythroid differentiation | Orkin & Zon, *Cell* (2008) [1]. |
| HBA2 | Erythroid | Hemoglobin subunit α2 | Canonical marker of erythroid differentiation | Orkin & Zon, *Cell* (2008) [1]. |
| GYPA | Erythroid | Glycophorin A | Erythroid membrane glycoprotein | Orkin & Zon, *Cell* (2008) [1]. |
| BPGM | Erythroid | 2,3-bisphosphoglycerate mutase | Erythroid metabolic enzyme | Orkin & Zon, *Cell* (2008) [1]. |
| ALAS2 | Erythroid | 5′-Aminolevulinate synthase 2 | Erythroid heme biosynthesis enzyme | Orkin & Zon, *Cell* (2008) [1]. |
| SLC4A1 | Erythroid | Band 3 anion exchanger | Mature erythrocyte membrane protein | Orkin & Zon, *Cell* (2008) [1]. |
| EPB42 | Erythroid | Erythrocyte membrane protein band 4.2 | Mature erythrocyte membrane protein | Orkin & Zon, *Cell* (2008) [1]. |
| RHCE | Erythroid | Rh blood group antigen | Mature erythroid marker | Human Cell Atlas Bone Marrow [2]. |
| MS4A1 (CD20) | B cell | B-cell surface antigen CD20 | Canonical mature B-cell marker | Human Cell Atlas Bone Marrow [2]. |
| CD79A | B cell | B-cell receptor signaling component | Canonical B-cell marker | Human Cell Atlas Bone Marrow [2]. |
| CD79B | B cell | B-cell receptor signaling component | Canonical B-cell marker | Human Cell Atlas Bone Marrow [2]. |
| CD3D | T cell | T-cell receptor CD3δ chain | Canonical T-cell marker | Human Cell Atlas Bone Marrow [2]. |
| CD3E | T cell | T-cell receptor CD3ε chain | Canonical T-cell marker | Human Cell Atlas Bone Marrow [2]. |
| CD2 | T cell | T-cell adhesion molecule | Canonical T-cell marker | Human Cell Atlas Bone Marrow [2]. |
| TRAC | T cell | T-cell receptor α constant region | Canonical T-cell marker | Human Cell Atlas Bone Marrow [2]. |
| LYZ | Myeloid | Lysozyme | Canonical monocyte/granulocyte marker | Human Cell Atlas Bone Marrow [2]. |
| MPO | Myeloid (granulocytic) | Myeloperoxidase | Granulocytic differentiation marker | Orkin & Zon, *Cell* (2008) [1]. |
| S100A8 | Myeloid | Calcium-binding protein S100A8 | Granulocyte/monocyte inflammatory marker | Human Cell Atlas Bone Marrow [2]. |
| S100A9 | Myeloid | Calcium-binding protein S100A9 | Granulocyte/monocyte inflammatory marker | Human Cell Atlas Bone Marrow [2]. |

**Supplementary Table S1.** Canonical lineage-associated genes were retained during differential expression analysis but excluded during the candidate autoantigen prioritization workflow to minimize enrichment of lineage-restricted differentiation markers in downstream HLA class II peptide prediction. Gene annotation was based on established hematopoietic lineage markers described by Orkin and Zon (Cell, 2008) [1]., the Human Cell Atlas Bone Marrow reference [2]., and the cell-type annotation strategy reported by Zhu et al. (Blood, 2021) [3]. This filtering step was intended to prioritize proteins broadly represented within the HSPC compartment rather than lineage-committed differentiation markers.

1. Orkin SH, Zon LI. Hematopoiesis: an evolving paradigm for stem cell biology. Cell. 2008 Feb 22;132(4):631-44. doi: 10.1016/j.cell.2008.01.025. PMID: 18295580; PMCID: PMC2628169.
2. Hay SB, Ferchen K, Chetal K, Grimes HL, Salomonis N. The Human Cell Atlas bone marrow single-cell interactive web portal. Exp Hematol. 2018 Dec;68:51-61. doi: 10.1016/j.exphem.2018.09.004. Epub 2018 Sep 21. PMID: 30243574; PMCID: PMC6296228.
3. Zhu C, Lian Y, Wang C, Wu P, Li X, Gao Y, Fan S, Ai L, Fang L, Pan H, Cheng T, Shi J, Zhu P. Single-cell transcriptomics dissects hematopoietic cell destruction and T-cell engagement in aplastic anemia. Blood. 2021 Jul 8;138(1):23-33. doi: 10.1182/blood.2020008966. PMID: 33763704; PMCID: PMC8349468.

**Supplementary Table S2. Public transcriptomic datasets included in the present study**

| GEO dataset | GEO accession(s) | Samples included | Disease status | Treatment status | Cell population | Sequencing platform | Role in the present study |
| --- | --- | --- | --- | --- | --- | --- | --- |
| GSE165870 | GSM5051834–GSM5051842 | Ctrl2, Ctrl7, Ctrl8, P18–P23 | Healthy controls (n = 3), acquired AA (n = 6) | Untreated | Lin⁻CD34⁺ HSPCs | Full-length bulk RNA-seq | Differential expression analysis |
| GSE145668 | GSM4323513–GSM4334968* | Ctrl1–Ctrl6, P1–P17 | Healthy controls (n = 6), acquired AA (n = 17) | Predominantly treatment-naïve† | Lin⁻CD34⁺ HSPCs, bone marrow T cells, peripheral blood T cells | Illumina HiSeq 4000 (GPL20301) | Cell-type prioritization of candidate genes relative to T cells |

**Supplementary Table S2.** Public transcriptomic datasets incorporated into the present study. Bulk RNA sequencing (GSE165870) and single-cell RNA sequencing (GSE145668).

* GSE145668 contains 11,456 single-cell transcriptomes, with individual GSM accessions corresponding to single-cell libraries generated from patients P1–P17 and healthy controls Ctrl1–Ctrl6.

† The original study included treatment-naïve patients for the primary single-cell characterization of HSPCs, while additional longitudinal samples collected after immunosuppressive therapy were generated for follow-up analyses. In the present study, GSE145668 was used exclusively as an independent cell-type prioritization resource to determine whether candidate genes identified by bulk RNA sequencing were preferentially expressed in HSPCs relative to T cells. It was not used as an independent validation cohort for disease-associated differential expression.
